# Determinants of Citizens’ Intention to Participate in Self-Led Contact Tracing: Cross-Sectional Online Questionnaire Study

**DOI:** 10.2196/56943

**Published:** 2024-10-30

**Authors:** Yannick Bernd Helms, Akke van der Meer, Rik Crutzen, José António Ferreira, Mirjam E E Kretzschmar, Aura Timen, Nora Hamdiui, Mart L Stein

**Affiliations:** 1 Centre for Infectious Disease Control (CIb) National Institute for Public Health and the Environment (RIVM) Bilthoven Netherlands; 2 Department of Health Promotion Care and Public Health Research Institute Maastricht University Maastricht Netherlands; 3 Department of Statistics, Informatics, and Modelling National Institute for Public Health and the Environment Bilthoven Netherlands; 4 Julius Center for Health Sciences and Primary Care University Medical Center Utrecht Utrecht University Utrecht Netherlands; 5 Department of Primary and Community Care Radboud Institute for Health Sciences Radboud University Medical Center Nijmegen Netherlands

**Keywords:** contact tracing, telemedicine, health services research, intention, public health surveillance, machine learning, cross-sectional study, online questionnaire, disease outbreaks, task shifting

## Abstract

**Background:**

Contact tracing (CT) is a key intervention to contain outbreaks of communicable diseases. During large-scale outbreaks, public health services may lack the resources required to perform CT effectively. One way of mitigating this issue is to shift some of the tasks in CT normally performed by public health services to cases and their contacts, supported by digital tools. We refer to this as “self-led CT.” However, while the effectiveness of the self-led CT inherently depends on the willingness and skills of citizens to participate, the determinants of citizens’ intention to participate in self-led CT are not yet fully understood.

**Objective:**

We aimed to identify determinants of Dutch citizens’ intention to participate in self-led CT and assess their potential for behavioral change, so as to identify “behavior change targets,” which may be used in the development and implementation of self-led CT to increase citizens’ intention to participate.

**Methods:**

In March 2022, we performed an online cross-sectional questionnaire study. The questionnaire was developed based on findings from a previous exploratory semistructured interview study and distributed among a Dutch consumer panel. Using all questionnaire items as potential predictors, we performed a random forest analysis to identify determinants of citizens’ intention to participate in self-led CT. We then performed an Agglomerative Hierarchical Cluster Analysis to identify groups of related determinants that may be considered overarching behavior change targets. Finally, we used Confidence Interval-Based Estimation of Relevance and calculated the Potential for Change Indices to compare the potential for behavioral change of the selected individual determinants and determinant clusters.

**Results:**

The questionnaire was completed by 3019 respondents. Our sample is representative of the Dutch population in terms of age, gender, educational level, and area of residence. Out of 3019 respondents, 2295 (76%) had a positive intention to participate in self-led CT. We identified 20 determinants of citizens’ intention that we grouped into 9 clusters. In general, increasing citizens’ trust in the digital tools developed for self-led CT has the highest potential to increase citizens’ intention, followed by increasing the belief that using digital tools makes participating in self-led CT easier, reducing privacy-related concerns, and increasing citizens’ willingness—and sense of responsibility—to cooperate in CT in general.

**Conclusions:**

Overall, Dutch citizens are positive toward participating in self-led CT. Our results provide directions for the development and implementation of self-led CT, which may be particularly useful in preparing for future, large-scale outbreaks.

## Introduction

Contact tracing (CT) is a key intervention to contain outbreaks of communicable diseases. CT is generally carried out by public health professionals (PHPs) who work for public health services (PHS). Although the precise execution of CT may depend on numerous factors, such as the specific pathogen at hand or the country or region where it is performed, it includes at least two crucial steps (1) contacting and interviewing individuals with a newly confirmed infection (ie, cases) to identify individuals who are at risk of having been infected because they had a physical interaction with the case during the case’s infectious period (ie, contacts) and (2) reaching out to the identified contacts and informing them of their exposure, their infection risk, and what measures should be taken to prevent further spread of the pathogen and severe disease (eg, testing, quarantine, vaccination, and post-exposure prophylaxis) [1,2].

The “traditional” approach to CT, as described, has been applied during numerous outbreaks involving different types of communicable pathogens [3]. During large-scale outbreaks, however, PHS may lack the human resources required to perform CT in a sufficiently complete (in terms of the proportion of at-risk contacts identified and notified) and timely manner to effectively reduce transmission and prevent (severe) disease among contacts [4,5]. This was, for example, often the case during the COVID-19 pandemic [6].

One solution to lower the burden on PHS and potentially accelerate the CT process is to shift some tasks in CT that are normally performed by PHPs to (a selection of) cases and contacts, with the help of digital tools (eg, a mobile app or website). We refer to this approach as “self-led CT.” For example, cases themselves could, supported through simple (audio-visual) guidelines and instructions, digitally make an overview of their contacts and collect their personal and their contacts’ data. Cases may potentially already do this before a PHP initiates the traditional CT process, for example, directly when they are tested for a given pathogen or when they receive their positive test result. Cases could then transfer the already collected data to case management software used by PHS when contacted by a PHP. In addition, cases may digitally inform (a selection of) their contacts of their exposure and of the measures that may be required to prevent further spread of the pathogen and severe disease. This could be done in advance of a PHP reaching out to their contacts, to speed up the notification process. Alternatively, cases could notify their contacts instead of a PHP, for example, when PHPs do not have time to reach out to all contacts, when cases do not want to share their contacts’ details with PHS, or when the PHPs cannot reach contacts.

Elements of self-led CT were (often implicitly) applied in many countries during the COVID-19 pandemic. In the Netherlands, for example, it became an important part of CT guidelines to ask relatively low-risk cases (eg, cases with relatively few vulnerable contacts) to notify their contacts themselves when PHS was too overwhelmed to perform CT for all cases [7]. This allowed PHPs to allocate more resources toward comparatively high-risk (clusters of) cases and contacts. However, the application of self-led CT was mainly necessity-driven and is not yet well understood in the context of large-scale outbreaks of pathogens that transmit via close direct or indirect contact between individuals (eg, via saliva, large droplets, or aerosols). Crucially, it is still largely unclear if and why citizens would not want to or want to use digital tools to perform the identification and notification of their contacts more autonomously under these circumstances. Previous research has mainly focused on understanding citizens’ intention to use other digital CT innovations, such as Bluetooth or GPS-based proximity tracing applications [8], or to participate in traditional (ie, PHP-led) CT. This is problematic since the “success” of self-led CT in practice inherently depends on the willingness and skills of citizens to participate. Research on citizens’ intention to participate in self-led CT is, therefore, urgently needed to prepare for future (large-scale) outbreaks where traditional CT and other alternatives (eg, proximity tracing apps) are not feasible, sufficient, or preferred.

To gain insight into the perspectives of citizens and the range of factors that influence their intention to participate in self-led CT, we previously conducted an exploratory semistructured interview study among 27 Dutch citizens during the COVID-19 pandemic [9]. We found that citizens were generally open to participating in self-led CT, but that their intention depends on a variety of factors, including their attitude toward CT in general, their trust in PHS and digital technologies, their capacity to digitally reach and inform their contacts, and the anticipated advantages of self-led CT (eg, having to spend less time talking to a PHP on the phone).

In this study, we built on our previous qualitative findings and distributed an online questionnaire among a consumer panel to recruit a representative sample of Dutch citizens. Our aim was to further enhance our understanding of citizens’ intention to participate in self-led CT and the determinants thereof. In addition, we investigated which clusters of determinants are suitable “behavior change targets,” in the sense that they may be leveraged in the development and implementation of self-led CT to increase citizens’ intention to participate. The research questions that guided this research were (1) “What are the determinants of citizens’ intention to participate in self-led CT?” and (2) “Which determinants can best be targeted to increase citizens’ participation in self-led CT?”

## Methods

### Study Design and Data Collection

In March 2022, we performed an online cross-sectional questionnaire study among Dutch citizens. We designed and implemented the study in line with the STROBE (Strengthening the Reporting of Observational Studies in Epidemiology) checklist for cross-sectional studies where applicable [10]. We distributed an online questionnaire among a Dutch consumer panel via a panel agency [11]. Members of the panel were informed about the study and what was expected of them upon invitation via the software system of the panel agency. We aimed to recruit a representative sample of Dutch citizens in terms of age (18 years or older), gender, educational level, and residential area within the Netherlands.

### Questionnaire

We developed an online questionnaire based on findings from a previous exploratory semistructured interview study where we used the reasoned action approach [12] to elicit citizens’ perspectives and beliefs related to their intention to participate in self-led CT in the context of CT for COVID-19 [9]. Questions were derived from qualitatively generated “themes” and formulated as statements to which respondents could respond on 5-point Likert scales. The questionnaire was piloted in-depth with 2 individuals recruited from the researchers’ personal networks and tested with colleagues from the Dutch National Institute for Public Health and the Environment (RIVM). After minor textual adjustments, the questionnaire was launched.

The questionnaire consisted of 4 main parts and contained 63 questions. In section one (5 questions), respondents were asked to fill out their sociodemographic characteristics, namely, age, gender, educational level, and residential area within the Netherlands. Respondents were also asked whether they had prior experience with CT for COVID-19 as a case or as a contact. In section two (16 questions), respondents first viewed a short information video (with textual support) explaining the traditional (ie, PHP-led rather than self-led) CT process for COVID-19. This was followed by statements related to participants’ perceptions of COVID-19 (eg, perceived severity), CT for COVID-19 (eg, perceived usefulness), and PHS in general (eg, trust in PHS). In sections 3 and 4 the principles of self-led CT—what is expected from citizens regarding the identification and notification of contacts—were explained to respondents through several images and textual explanations, specifically designed for this purpose. Section three (19 questions) contained statements about performing tasks in the identification and notification of contacts that are normally performed by PHPs (eg, anticipated advantages and challenges). Section four (20 questions) contained statements about the use of digital tools to perform these tasks. Participants’ intention to participate in self-led CT was assessed on a 5-point Likert scale via the statement “I am (Very willing; Willing; Neutral; Unwilling; Very unwilling) to digitally perform some steps in the CT-process myself.” Participants who indicated that they were “very willing” or “willing” received 2 follow-up statements with 3 answer options to assess the degree to which they would want to perform specific steps of the CT process (ie, contact identification) themselves or with help from a PHP (fully perform myself, partially perform myself and partially with a PHP, and completely leave up to the PHP).

The questionnaire and the images used to introduce respondents to self-led CT can be found in Multimedia Appendix 1. The questionnaire had a median completion time of 15 minutes.

### Data Analysis

We conducted the analysis in 4 steps. First, we described our sample using descriptive statistics. Second, we performed a random forest (RF) analysis, including all questionnaire items as potential predictors, to predict citizens’ intention to participate in self-led CT and to identify and select the determinants (ie, the strongest predictors) thereof. Third, we performed an Agglomerative Hierarchical Cluster Analysis (HCA) on the determinants selected in step 2, to identify “clusters” of determinants that may be considered as behavior change targets. Fourth, we used Confidence Interval-Based Estimation of Relevance (CIBER) plots and calculated the Potential for Change Indices (PCIs) to compare the behavioral change potential of the selected individual determinants and determinant clusters.

All analyses were performed in the RStudio Server (V2023.03.0; Posit). RF, HCA, and the behavior change assessment (using CIBER plots and PCIs) were performed using the R packages (R Core Team) “randomForest” [13], “factoextra” [14], and “behaviorchange” [15], respectively.

### Step 1: Descriptive Statistics

Descriptive statistics for all variables were computed for respondents who completed the online questionnaire (ie, provided answers to all questions). For categorical variables, we calculated proportions and for continuous variables the mean and SD. For consistency and to improve the interpretability of the results, we reversed the scale of several variables measured on a 5-point Likert scale so that 1 always represents the most desirable or favorable end of the spectrum and 5 the least desirable or favorable end (eg, questions 35-38 and 50-53 in Multimedia Appendix 1).

In the main text, we only report descriptives for the intention to participate in self-led CT and—for the subsample of respondents with a positive intention—preferences to participate in the identification and notification of contacts autonomously, or with help from a PHP. All other descriptives are reported in Table S1 in Multimedia Appendix 2.

### Step 2: Identifying Determinants of Citizens’ Intention to Participate in Self-Led CT

Using all questionnaire items as potential predictors of intention, we performed an RF analysis to identify determinants of citizens’ willingness to participate in self-led CT. RF is a machine learning algorithm that predicts an outcome based on a set of variables. RF yields a variable importance ranking (VIR) that reflects the relative contribution or “importance” of each variable to the accuracy of the predictions. The importance of a variable represents the increase in prediction error (measured in this study as the probability of misclassification, or “pmc”) resulting from the replacement of the variable’s value by a randomly chosen value drawn from the variable’s distribution. Thus, the greater the increase in the prediction error due to this “corruption,” the greater the importance of the variable. We chose RF because of its flexibility in dealing with many variables and its inherent ability to mimic the behavior of the data (eg, it automatically detects and models interaction effects and nonlinear relationships, and is robust to overfitting the data) [16].

We trained the RF model with the sample of respondents who completed the questionnaire. Since we were specifically interested in variables that determine a positive intention among citizens, we used a dichotomized intention variable as the outcome. Respondents who stated that they were “very willing” or “willing” to participate in self-led CT were classified as having a “positive” intention. Respondents who stated that they were “neutral,” “unwilling,” or “very unwilling” were classified as having a “neutral/negative” intention.

The RF model’s performance was measured by estimates of the pmc, sensitivity (sens, the probability of a correct prediction among “positive” individuals), and specificity (spec, the probability of a correct prediction among “neutral/negative” individuals). In addition, we determined the area under the receiver operating characteristic curve (AUC). The AUC is a commonly used metric to quantify the performance of classification models. Its value ranges between 0 and 1, with 0.5 corresponding to “random guessing” (“flip of a coin”) and 1 to perfect prediction. As a general guideline, AUC values of 0.8 and above can be considered “good” [17]. The AUC analysis is shown in Figure S1 in Multimedia Appendix 2.

We report the VIR, from which we visually identified the most important predictors (ie, determinants of citizens’ intention). As a loose guideline, we considered all predictors above the “cut” from where predictors vertically align on the left side of the VIR to be determinants of PHPs’ intention (predictors below this “cut” have little contribution to a model’s performance). We also report the model-based estimates of pmc, sens, spec, and AUC.

### Step 3: Identifying Clusters of Determinants

Since we derived our questionnaire items from qualitatively generated themes, we expected substantial overlap between determinants of citizens’ intention to participate in self-led CT. Although this does not affect the performance of the RF model, it does complicate the interpretation of the VIR, especially regarding the selection of behavior change targets. For example, it may not be sensible to focus only on 1 or 2 highly ranked determinants if these are strongly related to—or partially dependent on—other determinants. Rather, we suggest focusing on groups of highly ranked and related determinants. This approach is also grounded in behavioral theories that emphasize the interdependent nature of the beliefs that underpin behavioral intentions [12]. For this reason, the selection of behavior change methods is typically also linked to determinants at a more general level [18].

To identify groups, or “clusters,” of related determinants that may serve as overarching behavior change targets, we performed an HCA on a dissimilarity matrix whose entries are a function of 1 minus the Spearman correlation coefficient between pairs of determinants selected from the VIR 

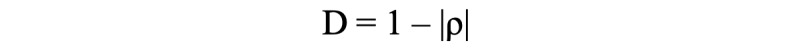



where D stands for dissimilarity, ρ is the Spearman correlation coefficient, and |ρ| its absolute value [19,20]. The Spearman correlation coefficient is an appropriate measure of similarity in the context of this study since it indicates how determinants “move together,” meaning that groups of clustered determinants relatively strongly depend on one another.

HCA uses an unsupervised machine learning algorithm that starts by considering all elements—in our case the determinants—to be separate clusters. Starting with the clusters that are most “similar” (ie, have the lowest D), pairs of clusters are then successively merged into larger clusters, until 1 large cluster remains. The results are visualized in a dendrogram, which is a tree-like representation of the clustering process containing information about the relationships between clusters at different levels of aggregation or similarity [20].

In addition to the measure of similarity or dissimilarity, the HCA algorithm uses a linkage rule to decide which clusters to merge and when. We chose the “average” linkage rule, which at each step of the clustering process merges the 2 clusters with the strongest average correlation between all determinants in the respective clusters.

We used the cophenetic correlation coefficient (CCC) to assess the overall quality of the HCA [21]. The CCC ranges between 0 and 1, with values closer to 1 indicating that the pairwise D between determinants in the dendrogram more closely resembles the unmodeled D from the original dissimilarity matrix. It serves as a goodness of fit indicator for the clustering process, with values closer to 1 indicating a better fit.

Determining an appropriate number of clusters (that is, at which point to “cut” the dendrogram so that the determinants within clusters are relatively similar to each other and dissimilar to determinants in the other clusters) is a somewhat subjective task that requires balancing the number of clusters and cluster complexity and parsimony. For the purposes of this study, we calculated the cluster “compactness” (ie, the maximum observed D between 2 determinants within any cluster) and the cluster “separation” (ie, the minimum observed D between 2 determinants from 2 different clusters) for all possible numbers of clusters (ie, 2 through 19). We then determined an “optimal” number of clusters by selecting that number for which the difference between compactness and separation is minimal. We tested this method and found that it works well (ie, identifies the right clusters quite often or quite closely) with simulated data on a known number of uncorrelated groups with varying numbers of correlated variables in them.

We report the cluster dendrogram, the CCC, and the plotted cluster compactness and separation for different numbers of clusters. We named the identified clusters to represent the individual determinants in them and report the average Spearman correlation between the determinants in each cluster (ρ_clus_). In addition, we calculated respondents’ mean cluster scores (ie, the average value of respondents’ responses to the questionnaire items or determinants in each respective cluster) and SDs (mean_clus_, SD_clus_).

### Step 4: Comparing the Potential for Behavioral Change of (Clusters of) Determinants

Finally, we compared the “potential for behavioral change” of the individual determinants identified in step 2 and the determinant clusters identified in step 3 using CIBER plots and PCIs [15,22,23]. Clusters of determinants have a high potential for behavioral change (ie, a high potential to increase citizens’ intention to participate in self-led CT on the population level) when they have a favorable univariate distribution, in the sense that there is sufficient “room for improvement” (ie, the mean score is not already at—or close to—the desired level), and a strong bivariate association with intention. We use bivariate associations to compare the potential for behavioral change of different determinants or clusters because these provide an unconditional (ie, independent from other determinants or clusters) impression of the potential effect of targeting a given determinant or cluster on citizens’ intention. To express the strength of bivariate associations we used Cohen *d* [24], which in this study is the standardized difference between the mean score of individuals with a positive intention and individuals with a neutral or negative intention toward participating in self-led CT, or

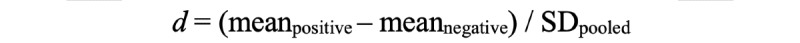



CIBER plots visualize the univariate distributions, the 95% CIs of the mean scores, and the 95% CIs of *d.* To aid the interpretation of the CIBER plots, we calculated PCIs for all individual determinants and determinant clusters. The PCI combines the room for improvement and *d* into a quantitative measure that can be used for comparative purposes, so that a higher PCI indicates a higher “change potential,” and vice versa. In this study, PCI was calculated as

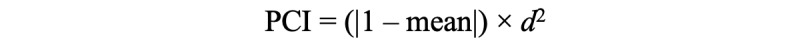



where |1 – mean| is the determinant or cluster level room for improvement and *d* is squared to penalize weaker associations. From this equation, it can be seen that when the mean score of a determinant or cluster is 1 (which is the most desirable or positive score, meaning that there is no room for improvement), or when *d* is 0 (meaning that there is no association between a given determinant or cluster and intention), then PCI is 0, meaning that the respective determinant or cluster has no potential to increase citizens’ intention to participate in self-led CT. We report the determinant and cluster level CIBER plots and PCIs.

### Ethical Considerations

This study was exempted from ethics approval in the Netherlands by the Medical Ethical Review Committee Utrecht (22-101/DB). Informed consent was obtained from each respondent prior to starting the online questionnaire. Respondents were explicitly asked to agree to the terms of the study through a digital informed consent form via the survey system of the panel agency, containing information and conditions of the study. Each respondent received a small monetary token of appreciation (provided by the panel agency) for their participation in the study.

## Results

### Sample Characteristics

In total, 3170 individuals started the online questionnaire. The questionnaire was completed by 3019 respondents (95% completion rate), who were included in our analyses. Sample characteristics are presented in Table 1. Characteristics of the Dutch population (provided by the panel agency) are also presented. Our sample was largely representative of the Dutch population in terms of all the presented demographic characteristics.

**Table 1:** Characteristics of the study population (N=3019).

Characteristics		Respondents, n (%)		Dutch population^a^
**Age (years)**				
	16-29	509 (16.9)	19%	
	30-44	726 (24.0)	23%	
	45-59	874 (28.9)	27%	
	60+	910 (30.1)	31%	
**Sex**				
	Male	1461 (48.4)	49%	
	Female	1548 (51.3)	51%	
	Other	10 (0.3)	N/A^b^	
**Educational level^a^**				
	Low	613 (20.3)	22%	
	Middle	1181 (39.1)	40%	
	High	1225 (40.6)	38%	
**Residential area**				
	Amsterdam, Rotterdam, The Hague, and suburbs (“Randstad”^c^)	543 (18.0)	16%	
	North Holland, South Holland, and Utrecht	919 (30.4)	29%	
	Friesland, Groningen, and Drenthe	290 (9.6)	10%	
	Overijssel, Gelderland, and Flevoland	562 (18.6)	21%	
	North Brabant, Limburg, and Zeeland	705 (23.4)	24%	
**Previous involvement in CT^d^**				
	Previously involved as a case	615 (20.4)	N/A	
	Previously involved as a contact	313 (10.4)	N/A	
	Experience with CT for other reason	34 (1.1)	N/A	
	No previous involvement	2057 (68.1)	N/A	

^a^Classification by Statistics Netherlands [25]. Only percentages are available and are provided by Norstat.

^b^N/A: not applicable.

^c^The “Randstad” is an urban agglomeration of cities and the most densely populated area in the Netherlands.

^d^CT: contact tracing.

### Descriptive Statistics

Out of 3019 respondents, 2295 (76%) had a positive intention to participate in self-led CT. Out of these, 1337 (58.3%) respondents indicated that, if they could choose, they would want to perform the identification of contacts completely autonomously (without any help from a PHP) and 1154 (50.3%) respondents would want to perform the notification of contacts completely autonomously. A complete overview of the descriptive statistics can be found in Table S1 of Multimedia Appendix 2.

### Determinants of Citizens’ Intention to Participate in Self-Led CT

**Figure 1:**
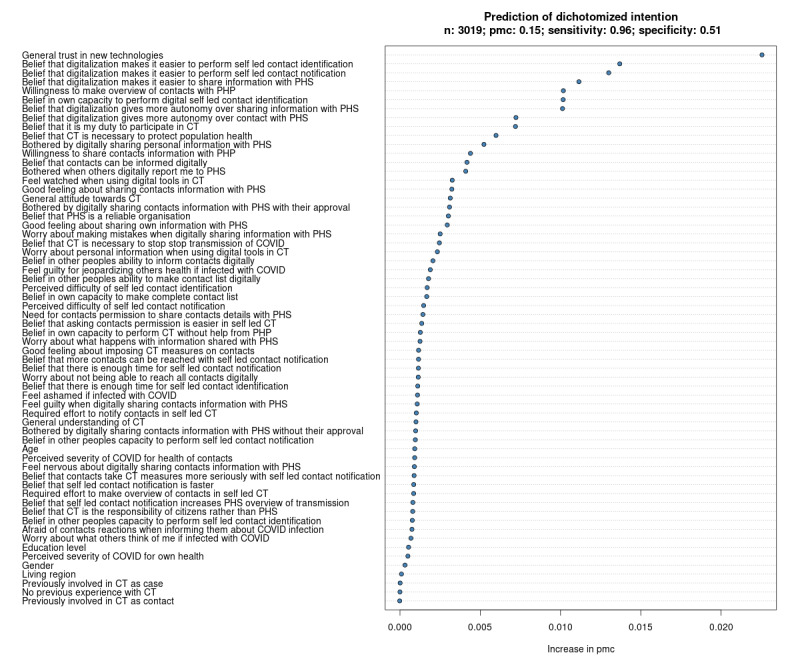
Variable importance ranking in relation to citizens’ intention to participate in self-led CT. CT: contact tracing; PHP: public health professional; PHS: public health service.

Using all questionnaire items as potential predictors, we built an RF model to predict citizens’ intention to participate in self-led CT and to identify the determinants thereof. The VIR is presented in Figure 1. Note that the value of “Increase in pmc” on the x-axis reflects the model’s absolute increase in pmc when a given variable is “corrupted,” as explained in the methods section. The model-based probability of a correct prediction among individuals with a positive intention (sens) is 0.96. The probability of a correct prediction among individuals with a neutral or negative intention (spec) is 0.51. The overall probability of a correct prediction is 0.85 (1-pmc). The RF model’s AUC is 0.89 (see Figure S1 in Multimedia Appendix 2).

The VIR indicates that citizens’ intention is determined by many determinants, rather than by a few top determinants. We, therefore, selected the top 20 predictors as determinants. The strongest determinant of intention was “General trust in new technologies.” Other important determinants were the belief that being able to digitally participate in self-led CT makes the identification and notification of contacts and sharing information with PHS easier, willingness to make an overview of contacts with a PHP, the belief in one’s own capacity to participate in self-led contact identification, and the belief that participating in self-led CT gives more autonomy over contact with PHS. The lowest-ranked predictor that we selected as a determinant is “Good feeling about sharing personal information with PHS.”

### Determinant Clusters

**Figure 2:**
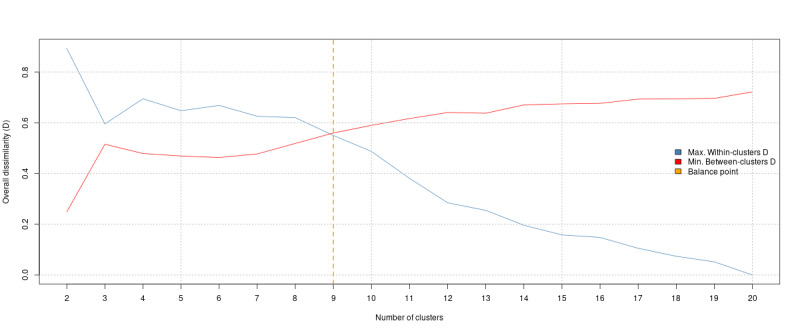
Cluster compactness and separation for different numbers of clusters. The y-axis shows the overall dissimilarity and the x-axis the number of clusters. Cluster compactness is indicated in blue and separation red. The optimal number of clusters is indicated in orange.

We performed an HCA on the Spearman correlation–based dissimilarity matrix of the top 20 determinants of citizens’ intention to participate in self-led CT that we selected from the VIR (Figure 1). Figure 2 shows the cluster compactness and separation for different numbers of clusters. The optimal number of clusters (ie, that number for which the difference between compactness and separation is minimal) was 9.

**Figure 3:**
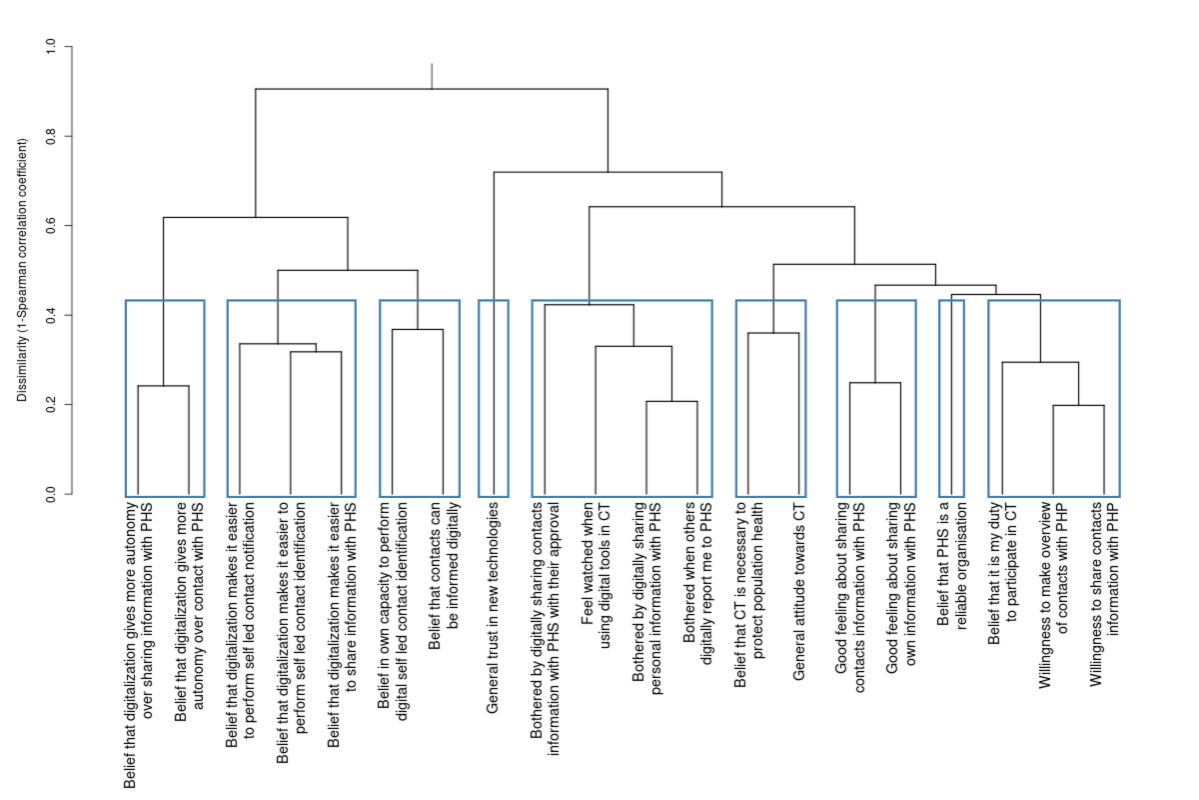
Cluster dendrogram of the top 20 determinants of citizens’ intention to participate in self-led CT. The y-axis represents D, determinants are displayed on the x-axis. The lower (clusters of) determinants are linked together in the dendrogram, the stronger the average correlation between them, and vice versa. Clusters are indicated by blue-grey borders (C1-9 from left to right). CT: contact tracing; PHP: public health professional; PHS: public health service.

Figure 3 shows the cluster dendrogram. The CCC was 0.95, meaning that the pairwise D between determinants in the dendrogram accurately reflects the unmodeled pairwise D from the original dissimilarity matrix, indicating a good fit with the data.

Cluster 1 (C1) was named “Increased autonomy in CT” and contains the beliefs that digitalization increases one’s autonomy over contact with PHS and over sharing information with PHS (mean_clus_ 2.12, SD_clus_ 0.76; ρ_clus_=0.76); C2 was named “Ease of digitally participating in CT” and includes the beliefs that digitalization makes the identification and notification of contacts and sharing information with PHS easier (mean_clus_ 2.20, SD_clus_ 0.70; ρ_clus_=0.67); C3 was named “Perceived ability to participate in self-led CT” and contains the beliefs that contacts can adequately be informed through digital means and the belief in one’s own capacity to participate in self-led CT (mean_clus_ 2.25, SD_clus_ 0.83; ρ_clus_=0.63); C4 contains only “General trust in new technologies” and was, therefore, also named as such; C5 was named “Privacy related concerns” and includes being bothered by sharing personal or contacts’ details with PHS, being bothered when others share your personal details with PHS, and feeling watched when using digital tools in CT (mean_clus_ 2.81, SD_clus_ 1.01; ρ_clus_=0.64); C6 was named “General attitude towards CT” and includes the belief that CT is necessary to protect population health and having a generally positive feeling about CT (mean_clus_ 2.03, SD_clus_ 0.79; ρ_clus_=0.64); C7 was named “Attitude towards sharing information with PHS” and contains having a good feeling about sharing personal and contacts’ information with PHS (mean_clus_ 2.87, SD_clus_ 0.92; ρ_clus_=0.75); C8 only contained the belief that PHS are reliable and was, therefore, named “Perceived reliability of PHS;” C9 was named “Felt responsibility and willingness to cooperate in CT” and includes the belief that it is one’s duty to participate in CT, and the willingness to make and overview of contacts—and share contacts’ details with a PHP (mean_clus_ 2.23, SD_clus_ 0.94; ρ_clus_=0.74). The dendrogram also shows that C1-3 are relatively strongly correlated, as are C5-9. C4 is a relatively isolated cluster.

### Comparison of the Behavioral Change Potential of (Clusters of) Determinants

**Figure 4:**
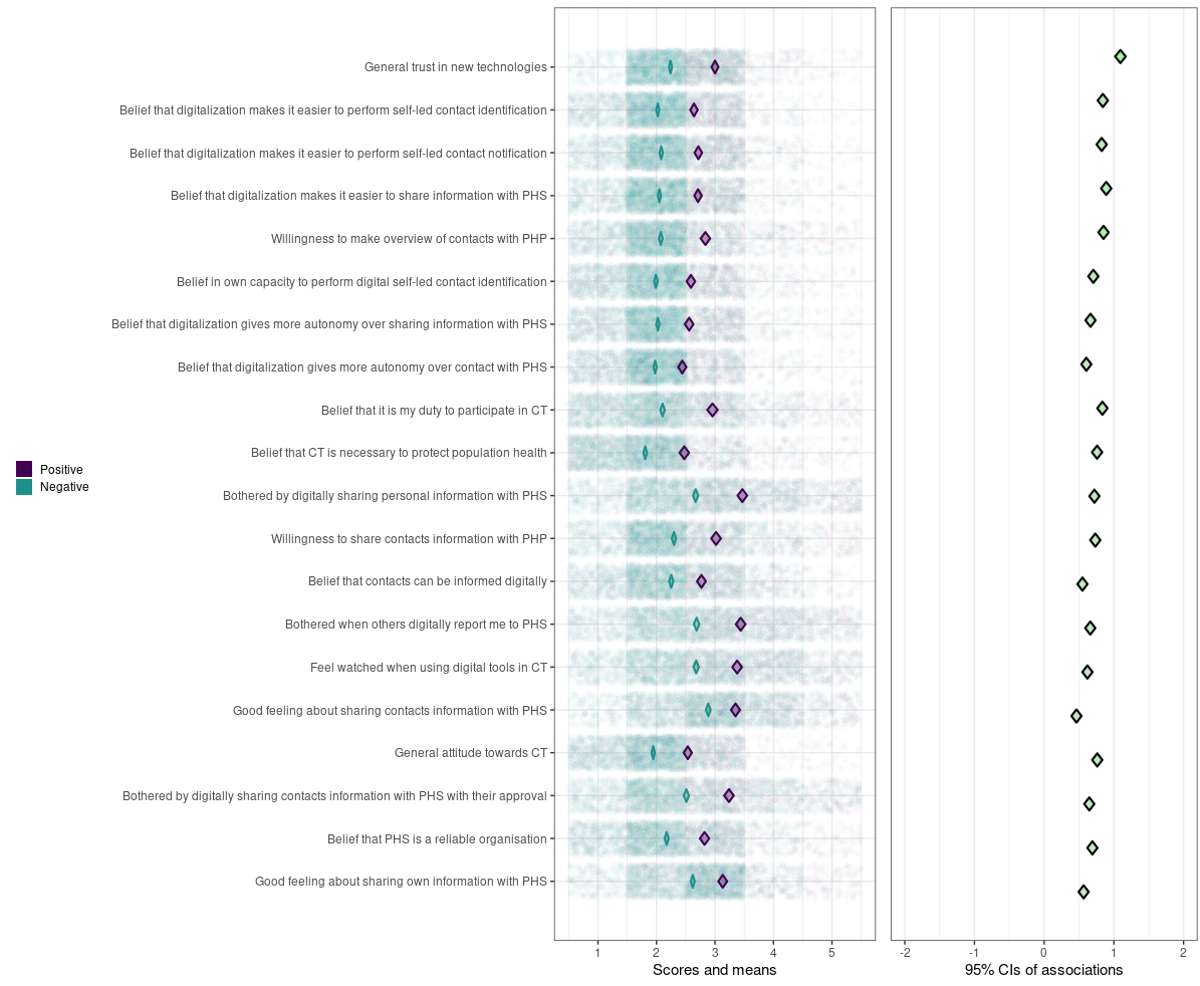
CIBER-plot for individual-level determinants of participation in self-led contact tracing. Population distributions and 95% CIs of the mean scores of individuals with a positive intention (green) and individuals with a negative intention (purple) are displayed in the left-hand panel; 95% CIs of the bivariate associations with intention (d) are displayed in the right-hand panel. Determinants are sorted in accordance with their VIR ranking displayed in Figure 1. CIBER: Confidence Interval-Based Estimation of Relevance; CT: contact tracing; PHP: public health professional; PHS: public health service; VIR: variable importance ranking.

**Figure 5:**
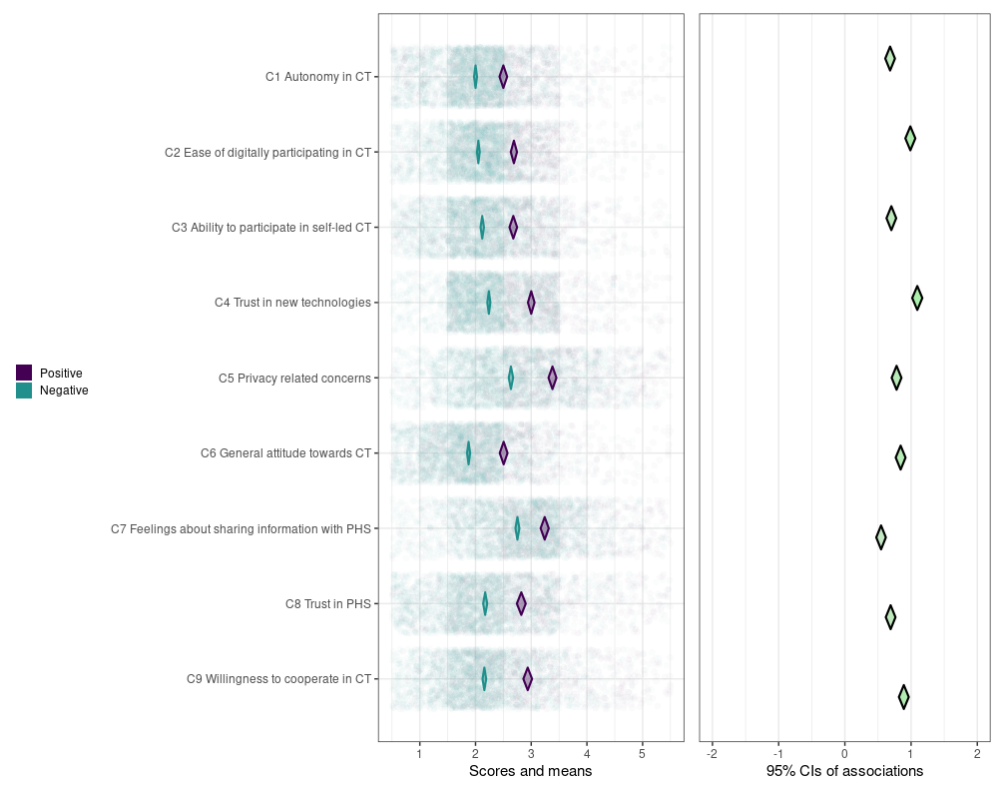
CIBER-plot for clustered determinants of participation in self-led contact tracing. Population distributions and 95% CIs of the mean scores of individuals with a positive intention (green) and individuals with a negative intention (purple) are displayed in the left-hand panel; 95% CIs of the bivariate associations with intention (d) are displayed in the right-hand panel. Clusters are sorted in accordance with the cluster dendrogram (Figure 3). CIBER: Confidence Interval-Based Estimation of Relevance; CT: contact tracing; PHS: public health service.

We created 2 CIBER plots; 1 for individual determinants (see Figure 4) and 1 for determinant clusters (see Figure 5). In the left-hand panels of Figures 4 and 5, the univariate distributions of the scores are visualized by transparent green dots for individuals with a positive intention and by purple dots for individuals with a neutral or negative intention to participate in self-led CT. The diamonds represent the 95% CIs of the mean scores of individuals with a positive and a neutral or negative intention. Note that lower scores always represent the “preferable/positive” side of the spectrum (eg, less worried and agreed to statement) and vice versa. The diamonds in the right-hand panel show the 95% CIs of *d*.

Most determinants and clusters have slightly right-skewed distributions, except for those related to privacy concerns and feelings about sharing information with PHS, which have a more centered distribution. For determinants or clusters with a more right-skewed distribution, individuals with a positive intention usually have a mean score of around 2, whereas individuals with a neutral or negative intention usually have a mean score around the scale median (between 2.5 and 3). For determinants or clusters with a more centered distribution, both positive and neutral or negative individuals tend to score around the scale median (between 2.5 and 3.5). This indicates that most determinants or clusters have at least some room for improvement. The value of *d* ranges between 0.55 and 1.10 for all individual determinants and determinant clusters, indicating that all determinants or clusters are also associated with citizens’ intention to participate in self-led CT.

To identify the determinants or clusters with the highest potential to increase citizens’ intention, we calculated PCIs (explained in more detail in the methods section). Trust in new technologies has the highest potential for behavioral change (PCI=1.70; see Table 2). Other individual determinants with a relatively high potential for behavioral change are being bothered by digitally sharing personal information with PHS (PCI=0.97), believing that digitalization makes it easier to share information with PHS (PCI=0.96), willingness to make an overview of contacts with a PHP (PCI=0.91), and believing that it is one’s duty to participate in CT (PCI=0.91). On the cluster level, C2 (ease of digitally participating in CT; PCI=1.20), C5 (privacy-related concerns; PCI=1.10), and C9 (felt responsibility and willingness to cooperate in CT; PCI=1.10) have the highest change potential, after trust in new technologies.

**Table 2:** Determinant selection table^a^.

Determinant or cluster		Individual determinant and cluster scores, mean (SD)	Association with intention, *d* (95% CI)	PCI^b^
**C1: Increased autonomy in CT^c^**				
	Belief that digitalization gives more autonomy over sharing information with PHS^d^	2.15 (0.84)	0.66 (0.58-0.75)	0.51
	Belief that digitalization gives more autonomy over contact with PHS	2.09 (0.79)	0.60 (0.52-0.69)	0.40
	Cluster	2.12 (0.76)	0.68 (0.60-0.77)	0.52
**C2: Ease of digitally participating in CT**				
	Belief that digitalization makes it easier to share information with PHS	2.21 (0.79)	0.89 (0.80-0.98)	0.96
	Belief that digitalization makes it easier to perform self-led contact notification	2.23 (0.82)	0.83 (0.74-0.91)	0.84
	Belief that digitalization makes it easier to perform self-led contact identification	2.17 (0.78)	0.84 (0.76-0.93)	0.83
	Cluster	2.20 (0.70)	0.99 (0.90-1.08)	1.20
**C3: Perceived ability to participate in self-led CT**				
	Belief in own capacity to perform digital self-led contact identification	2.13 (0.89)	0.71 (0.62-0.79)	0.56
	Belief that contacts can be informed digitally	2.38 (0.97)	0.55 (0.46-0.63)	0.41
	Cluster	2.25 (0.83)	0.70 (0.62-0.79)	0.62
**C4: General trust in new technologies**				
	General trust in new technologies	2.42 (0.77)	1.10 (1.01-1.18)	1.70
	Cluster	2.42 (0.77)	1.10 (1.01-1.18)	1.70
**C5: Privacy-related concerns**				
	Bothered by digitally sharing personal information with PHS	2.86 (1.16)	0.72 (0.64-0.81)	0.97
	Bothered when others digitally report me to PHS	2.87 (1.18)	0.66 (0.58-0.75)	0.82
	Feel watched when using digital tools in CT	2.85 (1.17)	0.62 (0.53-0.71)	0.71
	Bothered by digitally sharing contacts information with PHS with their approval	2.68 (1.17)	0.65 (0.56-0.73)	0.71
	Cluster	2.81 (1.01)	0.78 (0.69-0.87)	1.10
**C6: General attitude toward CT**				
	General attitude toward CT	2.09 (0.82)	0.76 (0.68-0.85)	0.63
	Belief that CT is necessary to protect population health	1.97 (0.93)	0.76 (0.67-0.84)	0.56
	Cluster	2.03 (0.79)	0.84 (0.76-0.93)	0.73
**C7: Attitude toward sharing information with PHS**				
	Good feeling about sharing own information with PHS	2.74 (0.93)	0.57 (0.48-0.65)	0.56
	Good feeling about sharing contacts information with PHS	3.00 (1.03)	0.46 (0.38-0.55)	0.43
	Cluster	2.87 (0.92)	0.55 (0.46-0.63)	0.56
**C8: Perceived reliability of PHS**				
	Belief that PHS is a reliable organization	2.33 (0.98)	0.69 (0.61-0.78)	0.63
	Cluster	2.33 (0.98)	0.69 (0.61-0.78)	0.63
**C9: Felt responsibility and willingness to cooperate in CT**				
	Willingness to make overview of contacts with PHP	2.26 (0.95)	0.85 (0.77-0.94)	0.91
	Belief that it is one’s duty to participate in CT	2.31 (1.09)	0.84 (0.75-0.92)	0.91
	Willingness to share contacts information with PHP	2.47 (1.03)	0.73 (0.65-0.82)	0.79
	Cluster	2.35 (0.94)	0.89 (0.80-0.98)	1.10

^a^Determinants in each cluster are sorted by PCI in descending order.

^b^PCI: Potential for Change Indices.

^c^CT: contact tracing.

^d^PHS: public health services.

## Discussion

### Main Findings

To accelerate CT and overcome resource limitations during large-scale outbreaks of communicable pathogens, PHS may need to shift tasks that are normally performed by PHPs to cases and their contacts through digital tools (eg, a website or mobile app). This approach, which we refer to as self-led CT, was (often implicitly) implemented by many countries during the COVID-19 pandemic and will likely become an important strategy again during future outbreaks. Nevertheless, very limited research has been performed on this subject and scientific guidance on the development and implementation of self-led CT is still scarce. Therefore, we aimed to identify determinants of citizens’ intention to participate in self-led CT and to determine which (clusters of) determinants may best be leveraged in the development and implementation of self-led CT to increase citizens’ intention to participate.

We identified 9 clusters with a total of 20 determinants of citizens’ intention that should be considered in the development and implementation of self-led CT. Out of these, we identified 4 clusters with a comparatively high “potential for change” (as expressed by the value of PCI), meaning that they still have “room for improvement” at the population level (ie, the determinant or cluster scores are not yet at the desired level) and are relatively strongly associated with citizens’ intention to participate in self-led CT. Increasing trust in (newly developed) digital tools for self-led CT has the highest potential to increase citizens’ intention to participate in self-led CT (PCI=1.70), followed by increasing the belief that using digital tools makes participating in self-led CT easier (PCI=1.20), reducing privacy-related concerns (PCI=1.10), and increasing citizens’ felt responsibility and willingness to participate in CT in general (PCI=1.10). Our combined results suggest that these (clusters of) determinants may be especially crucial with regard to increasing citizens’ intention to participate in self-led CT.

### Comparison With Literature

A large proportion (76%) of our sample had a positive intention to participate in self-led CT. Although this is an important finding that supports the implementation of self-led CT in practice, we would like to emphasize that this does not guarantee a similarly high level of adoption of self-led CT in practice. For example, our results are comparable to the results of a large multicountry survey (N=5995) on the acceptability of mobile proximity tracing apps that were conducted early in the COVID-19 pandemic, which found that 74.8% of their respondents would be willing to install a proximity tracing app on their phone [26]. Similarly, 1 study predicted that a 66% adoption rate of proximity tracing apps could be achieved in the Netherlands [27]. In practice, however, adoption rates of proximity tracing apps have proven to be much lower [28,29]. We believe that such an intention-behavior gap, which is broadly reported in previous literature when it comes to behavior change, may also (to some degree) reasonably be expected in the case of self-led CT. This should be considered when interpreting the results of this study.

Previous research on determinants of the adoption of digital tools for CT has mainly focused on self-monitoring tools or proximity tracing apps. Overall, such studies identified similar determinants to the ones identified in this study, such as privacy and security-related concerns [30,31], trust in new technologies for CT [32-35], and social responsibility [30,36]. Trust in the digital technologies used in CT emerges as a consistent predictor across studies, emphasizing its important role in shaping individuals’ intention to participate in self-led CT.

In contrast, 2 frequently discussed determinants of citizens’ intention to participate in digital CT that did not come up as important in this study were the perceived severity of COVID-19 for one’s personal health or for the health of vulnerable others [37,38]. We hypothesize that this may be related to the timing of our study, which was conducted toward the end of the COVID-19 pandemic. At that time, vaccinations were becoming available, measures to combat the pandemic were gradually being lifted, and society was reopening. It may be that this impacted citizens’ perception of the severity of COVID-19 or of the COVID-19 crisis in general [39]. Therefore, we believe that these factors should still be considered in the development and implementation of self-led CT.

Notably, a relatively large proportion of individuals with a positive intention to participate in self-led CT still indicated that they would want help (to some degree) from a PHP to perform the identification (41.7%) and notification (49.7%) of contacts. This corresponds with findings from a previous study in which we qualitatively explored citizens’ perspectives on the application of self-led CT [9]. In that study, we found that citizens mainly perceived self-led CT as a complementary approach to regular CT and that self-led CT should not replace the “traditional” CT process. Rather, there was a need for an integrated approach, tailored to the needs and preferences of citizens, with PHPs involved to support and guide cases and contacts in the CT process where needed.

Two other noteworthy determinants that we identified in this study were citizens’ general attitudes toward CT and their general willingness to cooperate in CT. The importance of these determinants with regard to citizens’ intention indicates that self-led CT may mainly be useful or attractive for citizens who are already positive about and motivated to participate in CT. This further illustrates the need for an integrated approach where PHPs may spend less time and resources in CT on motivated citizens—and more time on individuals who are hesitant toward participating in CT.

### Strengths and Limitations

Using a comprehensive combination of methods, we managed to identify the most relevant behavior change targets from a wide variety of candidates, that should be considered in the development and implementation of self-led CT to increase citizens’ intention to participate in self-led CT. The relevance of the identified behavior change targets with regards to citizens’ intention is also underscored by the prediction accuracy of our RF model, which had an AUC of 0.89 (see also Multimedia Appendix 2) Our findings are mainly relevant for the Dutch context since we base our findings on a representative sample of the Dutch population (in terms of age, gender, educational level, and residential area). Nevertheless, we believe that our results are also relevant for other highly digitalized Western high-income countries. That being said, we encourage similar research to be conducted in other countries. Another important strength is that we performed this study during the COVID-19 pandemic, meaning that our findings are particularly valuable and relevant for other future large-scale outbreaks with similar epidemiological characteristics.

Several limitations need to be considered. First, it is important to acknowledge that our recruitment strategy relied on an online consumer panel. This approach may have been a potential source of bias since panel members often show characteristics such as increased opinions, proactivity, and a desire to express their views [40,41]. In addition, since we used an online panel, our results may not be representative of less digitally skilled or oriented individuals. Second, even though citizens may have a generally positive intention to participate in self-led CT, their behavior in practice may differ depending on the specific context. For example, the number and types of contacts involved, or the specific CT measures that ought to be communicated, may considerably impact citizens’ intention to participate in self-led CT [9]. We are currently performing a separate study in which we are investigating this more in-depth. Third, although we successfully identified the most relevant behavior change targets that should be considered in the development and implementation of self-led CT, our results provide limited insights into the magnitude of the effect on citizens’ intentions that may be expected from targeting said behavior change targets. Although an assessment of this kind is beyond the scope and aims of this study, which were more exploratory in nature, we believe that this may be an interesting topic to explore in future research.

### Practical Implications

We performed an HCA to identify clusters of determinants that may be considered overarching behavior change targets to increase citizens’ intention to participate in self-led CT. The resulting dendrogram provides detailed insights into the relationships between determinants at different levels of aggregation or similarity, which may allow researchers or practitioners to select clusters at different levels, potentially also by applying criteria other than cluster compactness and separability (which is a data-driven approach to identify an “optimal” number of clusters), such as expertise and practical considerations. For example, Figure 3 suggests that an integrated approach may be required to leverage the behavior change potential of C4, C5, and C9, whereas C2 may be targeted relatively independently. The necessity for targeting multiple determinants or clusters is also underscored by the results of our RF analysis, which indicate that citizens’ intention is not determined by a few—but rather by a larger set of different determinants.

Although a variety of strategies may be chosen [42] to address the identified behavior change targets, we, based on our results, suggest to consider at least the general directions such as (1) adoption of a participatory or co-design approach to the development and implementation of (the digital tools for) self-led CT to enhance trust and user friendliness; (2) critically evaluating and minimalizing the amount of data and information asked from citizens in CT, and transparent communication about the purpose, processing, and handling of the collected data; (3) framing and communicating CT as a shared responsibility between PHS and citizen and emphasizing that citizens’ can play an important role in making CT more feasible and effective; (4) early communication (eg, directly when cases receive a positive test result) of the benefits of self-led CT for cases and PHS, such as faster notification of contacts and accelerated CT, which is better for outbreak control, and having to spend less time on the phone with a PHP; and (5) allowing cases to choose (where reasonable or possible) if and to what extent they would want to participate in self-led or conventional CT, or a combination thereof [43-46].

### Conclusions

Most Dutch citizens have a positive intention to participate in self-led digital CT. Important determinants of citizens’ intention include citizens’ trust in new technologies, the belief that digital tools make participating in self-led CT easier, privacy-related concerns, and perceived responsibility and willingness to cooperate in CT in general. Our results provide directions for the development and implementation of self-led CT in preparation for future (large-scale) outbreaks. In future research, we aim to investigate the applicability of self-led digital CT under different circumstances (eg, non-pandemic situations and less or more severe pathogens), the influence of contact network characteristics (eg, the number and type of contacts involved) on citizens’ intention to participate in (self-led) CT, and the effectiveness of self-led CT in practice.

## Appendix

Multimedia Appendix 1 Questionnaire.

Multimedia Appendix 2 Statistical analyses.

## Abbreviations

AUC: area under the receiver operating characteristic curve
CCC: cophenetic correlation coefficient
CIBER: Confidence Interval-Based Estimation of Relevance
CT: contact tracing
HCA: Agglomerative Hierarchical Cluster Analysis
PCI: Potential for Change Index
PHP: public health professional
PHS: public health services
RF: random forest
RIVM: National Institute for Public Health and the Environment
STROBE: Strengthening the Reporting of Observational Studies in Epidemiology
VIR: variable importance ranking
