# Supplementary material for: Determinants of Citizens’ Intention to Participate in Self-Led Contact Tracing: Cross-Sectional Online Questionnaire Study
Source: JMIR Public Health Surveill. 2024 Oct 30;10:e56943. doi: 10.2196/56943 (PMC11561431; doi:10.2196/56943)
Supplement: Multimedia Appendix 1 [file publichealth_v10i1e56943_app1.pdf]

## Appendix A. Questionnaire (translated from Dutch to English)

**Section 1.**

| |
|---|
| First, we would like to ask you a few personal questions. |

1. What is your age?

[…]

2. What is your gender?

[1] Male

[2] Female

[3] Other [specify]

3. What is the highest educational level that you have achieved?

[1] Primary school, junior high school or MBO 1

[2] MBO 2, 3, 4 or senior high school

[3] HBO or WO

4. Where do you live?

[1] Amsterdam, Rotterdam, The Hague, and suburbs

[2] North Holland, South Holland, and Utrecht

[3] Friesland, Groningen, and Drenthe

[4] Overijssel, Gelderland, and Flevoland

[5] North Brabant, Limburg, and Zeeland

**Section 2.**

**The next part of this survey is about contact tracing.**

When you have corona, it is important to quickly find out if people around you may also have been infected.

To this end, someone from public health services (named 'GGD' in the Netherlands) will carry out **contact tracing** with you.

**The goal of contact tracing** is to identifying people whom you have been close to whilst you were infected with the coronavirus. Those people will receive a message from the GGD that they may also have the coronavirus and receive information about what they can do to prevent further spread of the virus (e.g., stay at home, get tested)

5. Have you been called by the GGD before for contact tracing? You can give multiple answers to this question.

[1] Yes, because I had the coronavirus

[2] Yes, because I had been around someone who had the coronavirus

[3] Yes, other […]

[4] No

**The following statements are about how you think about the coronavirus. For each statement, click on the answer that suits you best.**

6. I think that the coronavirus can have [no - mild – somewhat severe - severe - very severe] consequences for my health
7. I think that the coronavirus can have [no - mild - somewhat severe - severe - very severe] consequences for the people I have been around to

**The following statements are about how you feel about contact tracing in general. For each statement, click on the answer that suits you best.**

8. I think that I have a [very good - good - not good or bad - bad - very bad] idea of what contact tracing entails
9. I think that it is [very good - good - not good or bad - bad - very bad] that contact tracing is carried out
10. I think that contact tracing is [very necessary – necessary – not necessary or unnecessary – unnecessary – very unnecessary] to control the spread of the coronavirus
11. I think that contact tracing is [very necessary – necessary – not necessary or unnecessary - unnecessary – very unnecessary] to protect (vulnerable) people

**Suppose you have tested positive for the coronavirus (which means that you have corona), and a GGD-employee calls you for contact tracing. Then...**

12. …I would find it [very pleasant – pleasant – not pleasant or bothersome – bothersome – very bothersome] to share personal information on the phone with the GGD-employee
13. …I would find it [very pleasant – pleasant – not pleasant or bothersome – bothersome – very bothersome] to share contact details of the people I have been around to on the phone with the GGD-employee

14. …I would find it [very pleasant – pleasant – not pleasant or bothersome – bothersome – very bothersome] that the people I have been around to may have to take measures (e.g., stay at home) because I report them to the GGD-employee
15. …I would be [very willing – willing – not willing or unwilling – unwilling – very unwilling] to make an overview of the people I have been around to together with the GGD-employee on the phone
16. …I would be [very willing – willing – not willing or unwilling – unwilling – very unwilling] to share the contact details of these people with the GGD-employee, so that the GGD-employee can inform these people about what measures they may need to take
17. …I would provide the contact details of the people I have been around to…

[1] …share with the GGD-employee

[2] …only share with the GGD-employee after these persons have given me permission

[3] …not share with the GGD-employee

**The following statements are about what you think about contact tracing and the GGD. For each statement, click on the answer that suits you best.**

18. I feel that it is my duty to participate in contact tracing [strongly agree - agree - neutral - disagree - strongly disagree]
19. I think that the GGD is a reliable organization [strongly agree - agree - neutral - disagree - strongly disagree]
20. I am concerned about what happens to the information I share with the GGD-employee on the phone [strongly agree - agree - neutral - disagree - strongly disagree]

**Section 3.**

**The next part of the questionnaire is about opportunities for you to carry out certain steps during contact tracing yourself, instead of with a GGD-employee. We would like to know if you could and would like to help the GGD more during contact tracing.**

**Suppose you tested positive for the coronavirus (which means that you have corona), and a GGD-employee calls you.**

**Step 1 would be: identifying your contacts**
The GGD-employee asks you to make an overview of the people you have been around to (your 'contacts'). These individuals may now also be at risk of being infected with the coronavirus. You make this overview on your own, instead of doing it together with the GGD-employee.

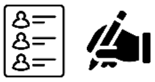

**Step 2 would be: notifying your contacts**
The GGD-employee asks you to inform the people you have been around to about what measures to take (e.g., stay at home) and what they should pay attention to (e.g., symptoms). You do this yourself, instead of the GGD-employee doing this.

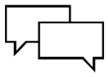

**The following statements are about how you think about carrying out certain steps yourself during contact tracing. For each statement, click on the answer that suits you best.**

21. In general, I find the execution of contact tracing...

[1] ...entirely the responsibility of GGD

[2] ...mainly the responsibility of the GGD

[3] ...partially the responsibility of the GGD and partially of citizens who have corona and the people they have been around to

[4] ...mainly the responsibility of the citizens who have corona and the people they have been around to

[5] ...entirely the responsibility of the citizens who have corona and the people they have been around to

**The following statements are about how you think about performing certain steps yourself during contact tracing. For each statement, click on the answer that suits you best.**

22. It seems [very easy – easy – not easy or difficult – difficult – very difficult] to me to make a list of the people I have been around to
23. It seems [very easy – easy – not easy or difficult – difficult – very difficult] to me to send (some of) the people I have been around to information about the measures they may need to take
24. I think that I can make a complete list of all the people I have been around to [strongly agree - agree - neutral - disagree - strongly disagree]
25. I think that I can do contact tracing all by myself, without contact with a GGD-employee [strongly agree - agree - neutral - disagree - strongly disagree]
26. It would take me [very little– little– not a little or a lot of – somewhat much – a lot of] effort to make a list of the people I have been around to

27. It would take me [very little – little – not a little or a lot of – somewhat much – a lot of] effort to inform the people I have been around to about the measures they may need to take
28. I think that it will go [much faster – faster – not faster or slower – slower – much slower] if I send the people I have been around to information about the measures they may need to take (instead of the GGD-employee doing this)
29. I think that the GGD has [much more – more – not more or less – less – much less] insight into the spread of the coronavirus if I inform the people I have been around to (instead of the GGD-employee doing this)
30. I think that people I have been around to will take the information about measures they may need to take [much more seriously – more seriously – not more or less seriously – less seriously – much less seriously] if I inform them (instead of the GGD-employee doing this)
31. I have **enough time** to make an overview of the people I have been around to [strongly agree - agree - neutral - disagree - strongly disagree]
32. If I would make an overview of the people I have been around to, I would have **more time** to ask these people if I can share their contact details with the GGD-employee [strongly agree - agree - neutral - disagree - strongly disagree]
33. I have **enough time** to inform the people I have been around to about the measures they may need to take (e.g., stay at home) [strongly agree - agree - neutral - disagree - strongly disagree]
34. I think I will **reach more people** if I send the people I have been around to information about the measures they may need to take (instead of the GGD-employee doing this) [strongly agree - agree - neutral - disagree - strongly disagree]

**The following statements are about your feelings regarding corona and how to carry out certain steps yourself during contact tracing. For each statement, click on the answer that suits you best.**

*'If I had corona, I would...'*

35. …feel ashamed [strongly agree - agree - neutral - disagree - strongly disagree]
36. …worry about what others think of me [strongly agree - agree - neutral - disagree - strongly disagree]
37. …feel guilty about possibly having jeopardized the health of others [strongly agree - agree - neutral - disagree - strongly disagree]
38. …be afraid of the reaction of the people I have been around to when I inform them about the measures they may need to take (e.g., having to stay at home) [strongly agree - agree - neutral - disagree - strongly disagree]

**The following statements are about others who contact tracing on their own. For each statement, click on the answer that suits you best.**

39. In general, I expect <u>others</u> to be able to make an overview of the people they have been around to [strongly agree - agree - neutral - disagree - strongly disagree]
40. In general, I expect that <u>others</u> will be able to send information about the measures to (some of) the people they have been around to [strongly agree - agree - neutral - disagree - strongly disagree]

**Section 4.**

**The last part of the questionnaire is about opportunities for you to <u>digitally</u> carry out certain steps during contact tracing yourself, instead of with a GGD-employee.**

Earlier, we asked you if you could and would like to help the GGD with contact tracing. Now, we are curious whether you could and would want to **digitally** perform certain steps in contact tracing **by yourself**. This could, for example, be done via a website (which you can open on your laptop/computer) and/or an app (which you can download on your phone). These so-called '**digital tools**' will be offered by the **GGD**.

**Suppose you tested positive for the coronavirus (which means that you have corona), and a GGD-employee calls you.**

**Step 1 would be: Contact identification**
The GGD-employee asks you to make a **digital overview** of the people you have been around to and who may now also be at risk of being infected with the coronavirus.

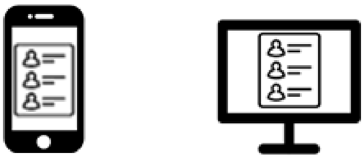

**Step 2 would be: Contact notification**
The GGD employee asks you to **digitally inform** the people you have been around to about the measures they can undertake (i.e., what they can do, what they should pay attention to and whether they might have to stay at home (quarantine)).

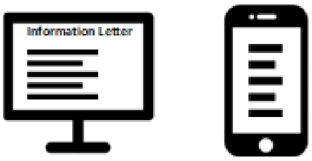


For each statement, click on the answer that suits you best.

41. In general, I have [a lot of trust – some trust – neutral – little trust – no trust] in new technologies
42. I am [very willing - willing – not willing or unwilling – unwilling – very unwilling] to digitally perform some tasks in contact tracing by myself

[58: If very willing - willing – not willing or unwilling]

43. If I test positive for the coronavirus (which means I have corona), then I am willing...
    [1] …to digitally make an overview of the people I have been around to **all by myself**
    [2] …to digitally make an overview of the people I have been around to **partially by myself** (and partially by the GGD-employee)
    [3] I prefer to make an overview of the people I have been around to **with the GGD-employee on the phone**

44. If I test positive for the coronavirus (which means I have corona), then I am willing...
    [1] …to digitally inform the people I have been around to about the measures they may need to take **all by myself**
    [2] … to digitally inform the people I have been around to about the measures they may need to take **partially by myself** (and partially by the GGD-employee)

[3] I prefer that the **GGD-employee** informs the people I have been around to about the measures they may need to take

**The following statements about reasons for digitally carrying out steps in contact tracing by yourself. For each statement, click on the answer that suits you best.**

***'By carrying out steps for contact tracing digitally...'***

45. I can [very easily – easily – not easily or difficult – difficult – very difficult] make an overview of the people I have been around to
46. I can [very easily – easily – not easily or difficult – difficult – very difficult] share information about myself and the people I have been around to with the GGD
47. I can [very easily – easily – not easily or difficult – difficult – very difficult] send the people I have been around to information about measures they may need to take
48. I can decide for myself what information I share with the GGD [strongly agree - agree - neutral - disagree - strongly disagree]
49. I can decide for myself when to contact the GGD, if necessary [strongly agree - agree - neutral - disagree - strongly disagree]

**The next statements are about reasons for NOT digitally carrying out steps in contact tracing by yourself. For each statement, click on the answer that suits you best.**

50. I feel guilty if I digitally report people I have been around to, to the GGD [strongly agree - agree - neutral - disagree - strongly disagree]
51. I feel nervous if I digitally report people I have been around to, to the GGD [strongly agree - agree - neutral - disagree - strongly disagree]
52. I'm afraid I won't be able to reach all the people I have been around to digitally [strongly agree - agree - neutral - disagree - strongly disagree]
53. I'm afraid that if I share information about myself and my contacts digitally, I may not be doing this properly [strongly agree - agree - neutral - disagree - strongly disagree]

**The following statements are about how you think about digitally carrying out certain steps yourself in contact tracing. For each statement, click on the answer that suits you best.**

54. I think I'm able to digitally make an overview of the people I have been around to [strongly agree - agree - neutral - disagree - strongly disagree]
55. I think I'm able to digitally inform all the people I have been around to about the measures they may need to take [strongly agree - agree - neutral - disagree - strongly disagree]
56. I have the feeling that I am being watched when I use digital tools in contact tracing [strongly agree - agree - neutral - disagree - strongly disagree]
57. I find it bothersome when someone digitally shares information about me with the GGD [strongly agree - agree - neutral - disagree - strongly disagree]
58. I find it bothersome to digitally share my personal information with the GGD [strongly agree - agree - neutral - disagree - strongly disagree]
59. I'm worried about what happens with my personal information when I do contact tracing using digital tools [strongly agree - agree - neutral - disagree - strongly disagree]
60. I find it bothersome to digitally share data of the people I have been around to, with the GGD without their permission [strongly agree - agree - neutral - disagree - strongly disagree]
61. I find it bothersome to digitally share data of the people I have been around to, with the GGD with their permission [strongly agree - agree - neutral - disagree - strongly disagree]

**The following statements are about <u>others</u> who carry out steps themselves during contact tracing. For each statement, click on the answer that suits you best.**

62. In general, I expect that <u>others</u> will be able to make a digital overview of the people they have been around to [strongly agree - agree - neutral - disagree - strongly disagree]
63. In general, I expect that <u>others</u> will be able to digitally send information about measures to (some of) the people they have been around to [strongly agree - agree - neutral - disagree - strongly disagree]

**Do you have any comments or questions? Let us know below.**
**This is the end of the survey. Thank you very much for your participation!**
