# Supplementary material for: Determinants of Citizens’ Intention to Participate in Self-Led Contact Tracing: Cross-Sectional Online Questionnaire Study
Source: JMIR Public Health Surveill. 2024 Oct 30;10:e56943. doi: 10.2196/56943 (PMC11561431; doi:10.2196/56943)

APPENDIX B. Supplementary statistical analyses

Table 1. Descriptive statistics for all questionnaire items for the whole sample and stratified for individuals with a (very) positive – and individuals with a neutral/negative intention.

**QUESTIONNAIRE SECTION 1: SOCIO-DEMOGRAPHIC AND PROFESSIONAL CHARACTERISTICS**			
Questionnaire item	Total sample (N=3019)	Positive intention (N=2295)	Neutral/negative intention (N=724)
**1.1. Age in years** (%)			
• 16 – 35	769 (25.5%)	585 (25.5%)	184 (25.4%)
• 36 – 55	1093 (36.2%)	831 (36.2%)	262 (36.2%)
• 56+	1157 (38.3%)	879 (38.3%)	278 (38.4%)
**1.2. Gender** (%)			
• Female	1548 (51.3%)	1164 (50.7%)	384 (53.0%)
• Male	1461 (48.4%)	1125 (49.0%)	336 (46.4%)
• Other	10 (0.3%)	6 (0.3%)	4 (0.6%)
**1.3. Educational level** (%)			
• Low	613 (20.3%)	417 (18.2)	196 (27.1%)
• Middle	1181 (39.1%)	881 (38.4%)	300 (41.4%)
• High	1225 (40.6%)	997 (43.4%)	228 (31.5%)
**1.4. Residential area (%)**			
• Amsterdam, Rotterdam, The Hague, and suburbs	543 (18.0%)	403 (17.6%)	140 (19.3%)
• North Holland, South Holland, and Utrecht	919 (30.4%)	713 (31.1%)	206 (28.5%)
• Friesland, Groningen, and Drenthe	290 (9.6%)	220 (9.6%)	70 (9.7%)
• Overijssel, Gelderland, and Flevoland	562 (18.6%)	435 (19.0%)	127 (17.5%)
• North Brabant, Limburg, and Zeeland	705 (23.4%)	524 (22.8%)	181 (25.0%)

**1.5. Previous involvement in CT** (%)			
• Previously involved as a case	615 (20.4%)	480 (20.9%)	135 (18.6%)
• Previously involved as a contact	313 (10.4%)	248 (10.8%)	65 (9.0%)
• Experience with CT for other reason	34 (1.1%)	25 (1.1%)	9 (1.2%)
• No previous involvement	2057 (68.1%)	1604 (69.9%)	525 (72.5%)
**QUESTIONNAIRE SECTION 2: CT FOR COVID**			
Questionnaire item	Total sample (N=3019)	Positive intention (N=2295)	Neutral/negative intention (N=724)
**2.1. Perceived severity of COVID for own health;** M (SD)			
• Not severe [1] – very severe [5]	2.69 (0.99)	2.76 (0.98)	2.49 (1.01)
**2.2. Perceived severity of COVID for health of contacts**; M (SD)			
• Not severe [1] – very severe [5]	2.88 (0.97)	2.97 (0.95)	2.59 (0.99)
**2.3. General understanding of CT**; M (SD)			
• Very good [1] – very bad [5]	2.02 (0.71)	1.95 (0.66)	2.25 (0.83)
**2.4. General attitude towards CT**; M (SD)			
• Very positive [1] – very negative [5]	2.09 (0.82)	1.94 (0.70)	2.53 (0.97)
**2.5. Belief that CT is necessary to stop transmission of COVID**; M (SD)			
• Definitely yes [1] – definitely no [5]	2.31 (0.98)	2.16 (0.87)	2.78 (1.15)
**2.6. Belief that CT is necessary to protect population health**; M (SD)			
• Definitely yes [1] – definitely no [5]	1.97 (0.93)	1.81 (0.79)	2.48 (1.12)
**2.7. Good feeling about sharing own information with PHS**; M (SD)			
• Very good [1] – very bad [5]	2.74 (0.93)	2.62 (0.86)	3.13 (1.05)

**2.8. Good feeling about sharing contacts information with PHS**; M (SD)<br>• Very good [1] – very bad [5]	3.00 (1.03)	2.88 (0.98)	3.35 (1.07)
**2.9. Good feeling about imposing CT measures on contacts;** M (SD)<br>• Very good [1] – very bad [5]	3.05 (1.11)	2.96 (1.09)	3.33 (1.13)
**2.10. Willingness to make overview of contacts with PHP;** M (SD)<br>• Very willing [1] – very unwilling [5]	2.26 (0.95)	2.07 (0.81)	2.84 (1.12)
**2.11. Willingness to share contacts information with PHP;** M (SD)<br>• Very willing [1] – very unwilling [5]	2.47 (1.03)	2.30 (0.92)	3.02 (1.15)
**2.12. Need for contacts permission to share contacts details with PHS;** M (SD)<br>• Definitely yes [1] – definitely no [5]	1.78 (0.65)	1.70 (0.61)	2.03 (0.71)
**2.13. Belief that it is my duty to participate in CT;** M (SD)<br>• Definitely yes [1] – definitely no [5]	2.31 (1.09)	2.10 (0.95)	2.96 (1.23)
**2.14. Belief that PHS is a reliable organization;** M (SD)<br>• Very reliable [1] – very unreliable [5]	2.33 (0.98)	2.17 (0.88)	2.82 (1.09)
**2.15. Worry about what happens with information shared with PHS**<br>• Not worried [1] – very worried [5]	3.12 (1.09)	3.03 (1.08)	3.41 (1.07)
**2.16. Belief that CT is the responsibility of citizens rather than PHS;** M (SD)<br>• Definitely yes [1] – definitely no [5]	3.33 (0.95)	3.33 (0.90)	3.33 (1.10)

QUESTIONNAIRE SECTION 3: SELF-LED CT			
Questionnaire item	Total sample (N=3019)	Positive intention (N=2295)	Neutral/negative intention (N=724)
**3.1. Perceived difficulty of self-led contact identification;** M (SD)<br>• Very easy [1] – very difficult [5]	2.28 (0.88)	2.22 (0.83)	2.47 (1.00)
**3.2. Perceived difficulty of self-led contact notification;** M (SD)<br>• Very easy [1] – very difficult [5]	2.43 (0.93)	2.37 (0.89)	2.64 (1.02)
**3.3. Belief in own capacity to make complete contact list;** M (SD)<br>• Definitely capable [1] – Definitely not capable [5]	2.26 (0.95)	2.16 (0.91)	2.55 (1.03)
**3.4. Belief in own capacity to perform CT without help from PHS;** M (SD)<br>• Definitely capable [1] – Definitely not capable [5]	2.57 (1.03)	2.54 (1.01)	2.65 (1.09)
**3.5. Required effort to make overview of contacts in self-led CT;** M (SD)<br>• Small effort [1] – large effort [5]	2.33 (0.87)	2.26 (0.82)	2.53 (0.99)
**3.6. Required effort to notify contacts in self-led CT;** M (SD)<br>• Small effort [1] – large effort [5]	2.54 (0.96)	2.48 (0.93)	2.72 (1.02)
**3.7. Belief that self-led contact notification is faster;** M (SD)<br>• Definitely yes [1] – definitely no [5]	2.30 (0.91)	2.25 (0.87)	2.45 (1.01)

**3.8. Belief that self-led contact notification increases PHS overview of transmission;** M (SD)<br>• Definitely yes [1] – definitely no [5]	3.06 (1.04)	3.03 (1.06)	3.17 (1.00)
**3.9. Belief that contacts take CT measures more seriously with self-led contact notification;** M (SD)<br>• Definitely yes [1] – definitely no [5]	2.88 (0.86)	2.87 (0.86)	2.91 (0.88)
**3.10. Belief that there is enough time for self-led contact identification;** M (SD)<br>• Definitely yes [1] – definitely no[5]	2.28 (0.93)	2.19 (0.87)	2.57 (1.05)
**3.11. Belief that asking contacts permission is easier in self-led CT;** M (SD)<br>• Definitely yes [1] – definitely no[5]	2.43 (0.89)	2.35 (0.85)	2.70 (0.96)
**3.12. Belief that there is enough time for self-led contact notification;** M (SD)<br>• Definitely yes [1] – definitely no[5]	2.39 (0.95)	2.30 (0.90)	2.66 (1.05)
**3.13. Belief that more contacts can be reached with self-led contact notification;** M (SD)<br>• Definitely yes [1] – definitely no[5]	2.61 (0.97)	2.56 (0.95)	2.77 (1.02)
**3.14. Feel ashamed if infected with COVID;** M (SD)<br>• Definitely no [1] – definitely yes [5]	1.87 (1.02)	1.83 (0.99)	2.00 (1.09)
**3.15. Worry about what others think of me if infected with COVID;** M (SD)<br>• Definitely no [1] – definitely yes [5]	1.94 (1.05)	1.92 (1.05)	2.00 (1.05)

**3.16. Feel guilty for possibly jeopardizing others' health if infected with COVID;** M (SD)<br>• Definitely no [1] – definitely yes [5]	3.11 (1.24)	3.20 (1.23)	2.83 (1.22)
**3.17. Afraid of contacts reactions when informing them about COVID infection;** M (SD)<br>• Definitely no [1] – definitely yes [5]	2.39 (1.11)	2.39 (1.12)	2.37 (1.09)
**3.18. Belief in other people's capacity to perform self-led contact identification;** M (SD)<br>• Definitely capable [1] – Definitely not capable [5]	2.30 (0.82)	2.25 (0.77)	2.46 (0.94)
**3.19. Belief in other people's capacity to perform self-led contact notification;** M (SD)<br>• Definitely capable [1] – Definitely not capable [5]	2.41 (0.85)	2.36 (0.82)	2.57 (0.93)
**QUESTIONNAIRE SECTION 4: SELF-LED CT**			
Questionnaire item	Total sample (N=3019)	Positive intention (N=2295)	Neutral/negative intention (N=724)
**4.1. General trust in new technologies;** M (SD)<br>• High trust [1] – Low trust [5]	2.42 (0.77)	2.24 (0.65)	3.00 (0.81)
**4.2. Belief that digitalization makes it easier to perform self-led contact identification;** M (SD)<br>• Definitely yes [1] – definitely no [5]	2.17 (0.78)	2.02 (0.68)	2.64 (0.89)
**4.3. Belief that digitalization makes it easier to share information with PHS;** M (SD)<br>• Definitely yes [1] – definitely no [5]	2.21 (0.79)	2.05 (0.69)	2.71 (0.89)

**4.4. Belief that digitalization makes it easier to perform self-led contact notification;** M (SD)<br>• Definitely yes [1] – definitely no [5]	2.23 (0.82)	2.08 (0.73)	2.72 (0.89)
**4.5. Belief that digitalization gives more autonomy over sharing information with PHS;** M (SD)<br>• Definitely yes [1] – definitely no [5]	2.15 (0.84)	2.02 (0.72)	2.56 (1.02)
**4.6. Belief that digitalization gives more autonomy over contact with PHS;** M (SD)<br>• Definitely yes [1] – definitely no [5]	2.09 (0.79)	1.98 (0.69)	2.44 (0.96)
**4.7. Feel guilty when digitally sharing contacts information with PHS;** M (SD)<br>• Definitely no [1] – definitely yes [5]	2.89 (1.12)	2.81 (1.10)	3.16 (1.15)
**4.8. Feel nervous about digitally sharing contacts information with PHS;** M (SD)<br>• Definitely no [1] – definitely yes [5]	2.83 (1.13)	2.79 (1.14)	2.97 (1.11)
**4.9. Worry about not being able to reach all contacts digitally;** M (SD)<br>• Definitely no [1] – definitely yes [5]	3.01 (1.12)	3.01 (1.11)	3.02 (1.14)
**4.10. Worry about making mistakes when digitally sharing information with PHS;** M (SD)<br>• Definitely no [1] – definitely yes [5]	2.63 (1.06)	2.55 (1.04)	2.91 (1.08)
**4.11. Belief in own capacity to perform digital self-led contact identification;** M (SD)<br>• Definitely capable [1] – Definitely not capable [5]	2.13 (0.89)	1.99 (0.79)	2.59 (1.01)

**4.12. Belief that contacts can be informed digitally;** M (SD)<br>• Definitely yes [1] – definitely no [5]	2.38 (0.97)	2.25 (0.90)	2.77 (1.05)
**4.13. Feel watched when using digital tools in CT;** M (SD)<br>• Definitely no [1] – definitely yes [5]	2.85 (1.17)	2.68 (1.13)	3.38 (1.14)
**4.14. Bothered when others digitally report me to PHS;** M (SD)<br>• Definitely no [1] – definitely yes [5]	2.87 (1.18)	2.69 (1.14)	3.44 (1.14)
**4.15. Bothered by digitally sharing personal information with PHS;** M (SD)<br>• Definitely no [1] – definitely yes [5]	2.86 (1.16)	2.67 (1.10)	3.47 (1.13)
**4.16. Worry about personal information when using digital tools in CT;** M (SD)<br>• Definitely no [1] – definitely yes [5]	3.10 (1.18)	2.94 (1.17)	3.60 (1.09)
**4.17. Bothered by digitally sharing contacts information with PHS without their approval;** M (SD)<br>• Definitely no [1] – definitely yes [5]	3.61 (1.16)	3.52 (1.18)	3.87 (1.07)
**4.18. Bothered by digitally sharing contacts information with PHS with their approval;** M (SD)<br>• Definitely no [1] – definitely yes [5]	2.68 (1.17)	2.51 (1.12)	3.24 (1.13)
**4.19. Belief in other people's ability to make contact list digitally;** M (SD)<br>• Definitely capable [1] – Definitely not capable [5]	2.35 (0.81)	2.28 (0.77)	2.58 (0.90)

**4.20. Belief in other people's ability to inform contacts digitally;** M (SD)<br>• Definitely capable [1] – Definitely not capable [5]	2.40 (0.82)	2.32 (0.78)	2.65 (0.90)
**QUESTIONNAIRE SECTION 5: NEED FOR PHS SUPPORT**			
Questionnaire item	Total sample (N=3019)	Positive intention (N=2295)	Neutral/negative intention (N=724)
**5.1. Willingness to perform contact identification fully autonomously, or with help from a PHP (%)**<br>• Fully autonomously<br>• Need some help<br>• Want to leave completely to PHP	N/A	1337 (58.2%)<br>809 (35.2%)<br>149 (6.5%)	N/A
**5.2. Willingness to perform contact notification fully autonomously, or with help from a PHP (%)**<br>• Fully autonomously<br>• Need some help<br>• Want to leave completely to PHP	N/A	1154 (50.3%)<br>871 (38.0%)<br>270 (11.8%)	N/A

*N/A = Not applicable

**M (SD)= Mean (Standard deviation)

Figure 1. Analysis of the area under the receiver operator curve (AUC) of the random forest model with all questionnaire items as predictors. The value of AUC ranges between 0 and 1, where 1 indicates perfect prediction and 0.5 indicates random guessing. A value of 0.89 can be considered as very good. The Figure also shows the variability of SENS and SPEC (1-fpr) for different cutoff values(c).

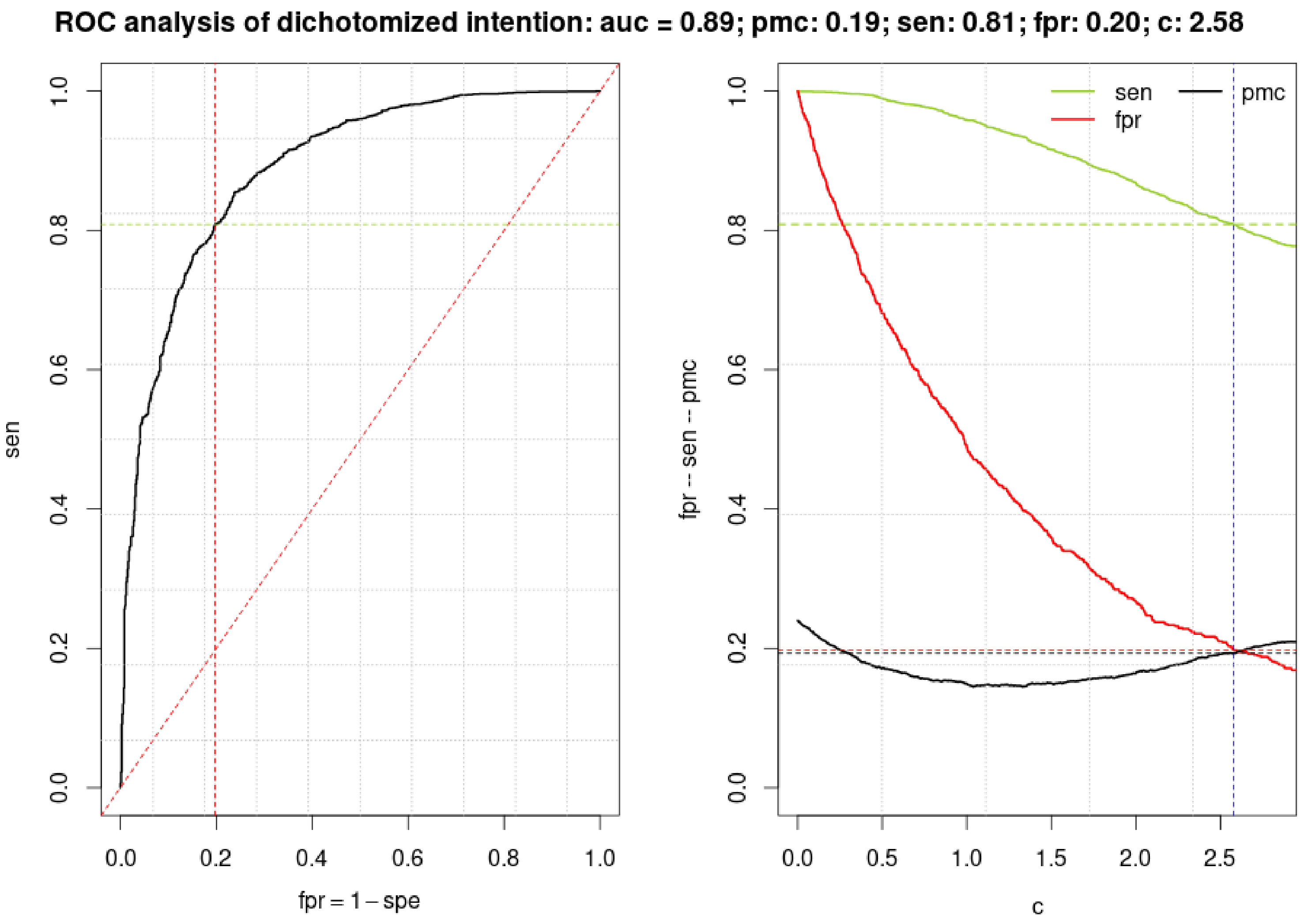

Supplement: Multimedia Appendix 2 [file publichealth_v10i1e56943_app2.pdf]
